# Determining management strategies for the Sarikum Nature Protection Area

**DOI:** 10.1007/s10661-015-4302-3

**Published:** 2015-02-13

**Authors:** Sevgi Öztürk

**Affiliations:** Faculty of Engineering and Architecture, Landscape Architecture Department, Kastamonu University, 37200 Kastamonu, Turkey

**Keywords:** Protection-usage balance, Wetland, Management strategies, R′WOT Analysis

## Abstract

In recent years, many environmental problems have become important factors in promoting the economic need to develop tourist activity: climate change such as energy wars, increasing hunger and aridity, population increases in urban areas, excessive and unthinking use of natural resources, difficult international relations, economic competition, and increasing environmental stress. Trends in global tourism have changed with changes in culture and our attitude to nature. Changes in both the profile and consumption patterns of tourists have called for the need to balance the use of natural and cultural assets with the need to adequately protect them. In this study, the Sarikum Nature Protection Area (SNPA) was selected as a case study because of its significance as a Turkish wetland area and the variety of different ecosystems coexisting within it. The study focussed on management strategies, but also provides a broader strategy for an area that currently has no management plan. Strengths and weaknesses, opportunities and threats (SWOT) analyses of the area were gathered and analyzed using R′WOT analysis (ranking + SWOT), a multi-criteria assessment method, in order to determine strategies, obtain the participation of interest groups, and assess their opinions and attitudes. The analysis showed the following: the rich biological diversity and the existence of endemic species were the reserve’s most significant strength; the presence of natural areas in surrounding regions was the most significant opportunity; the shortage of infrastructure and lack of legal regulation of ecotourism was the most significant weakness; and the lack of a management plan was the most immediate threat.

## Introduction

Due to the fact that the uncontrolled use of natural resources, the ecological balance has been damaged (Akbulak and Cengiz 2014; Yücel and Babuş 2005). In order to balance earth’s ecology, natural resources and species must be preserved and developed in a sustainable way. As it is known, for implementing this preservation, governments have their own legislations about natural resources’ protection in local, regional, and national scales. They determine valuable, unique, and rich environments and call them protected areas. These areas are described by The International Union for the Conservation of Nature as the areas which need to be managed by laws and planning instruments and preserved because of their naturally and culturally distinctive characteristics and biological diversity in order to sustain their existence to the future (International Union for the Conservation of Nature 1994).

The main reasons which give rise to deterioration of life environments of living creatures, alteration of landscape, and the rise of land use density in protected areas are known as economic and social activities. Nowadays, tourism activities to protected areas and natural sites come into question with their damages to the environment. Therefore, sustainability and its implementation became very significant for preserving natural sites (Akbulak and Cengiz 2014). In this context, different tourism types came into agenda for keeping tourism activities in these sites without damaging the nature.

Advances in the study of tourism have led to the development of concepts such as “rural tourism,” “ecotourism,” and “sustainable tourism.” Ecotourism which is focused in this study’s context, basically purposes to protect natural and cultural richness while local people’s economic welfare increases. Therefore, the importance of ecotourism activity raised in many cases (Cengiz 2009; Day and Cai 2012). Ecotourism is a reaction contrary to mass tourism, and is not limited to a specific season, covers rural and cultural tourism elements, and is considered the most suitable type of tourism for development in environmentally sensitive areas (Kaypak 2012). According to the Ecotourism Society (1990), ecotourism is “sensitive travel which protects the environment and observes the welfare of local people and which is made in natural areas.” Ecotourism does not have a single, universally accepted definition or a well-defined market. According to many scientists, it is based on an approach that assures, protects, and observes the sustainability and social and cultural integrity of natural resources, while supporting the economic development of local people (Akay and Zengin 2012; Boo 1991; Eagles 1995; Wight 1993). Ecotourism includes concepts of social responsibility, economic efficiency, and ecological sensitivity (Cater 1994; Rahemtulla and Wellstead 2001). Additionally, it contributes to the understanding of the need to protect nature in both the visitors and local people. However, as in other types of tourism, ecotourism has both pros and cons. Ecotourist activities may not always bring the desired results in terms of net benefits to the locality visited. For instance, the carrying capacity of natural areas may be exceeded and environmental problems, cultural degeneration, and the overconsumption of renewable energy sources may arise. Whenever ecotourism opportunities are developed, it is necessary to create and implement appropriate management plans to ensure effective protection of the area concerned.

In this study, we suggest that the Sarikum Nature Protection Area (SNPA) qualifies as a protected area and that participatory management strategies are developed there that recognize its sensitive ecosystems and high visitor potential. It is an important wetland area in Turkey. Wetland areas are significant in an ecosystems context and are essential to protect natural balance and biodiversity. Furthermore, they provide food, breeding sites, and a sheltered environment for many species and therefore have global importance (Öztürk et al. 2012).

## Methods

The province of Sinop, located in the West Black Sea Region, contains one natural area under protection, three natural parks, and three natural monuments, according to the Law of National Parks number 2873 (Republic of Turkey Ministry of Forestry and Water 10. Regional Direction 2013). The study methodology comprises data collection and an R′WOT analysis (Yilmaz 2006; Öztürk and Tönük 2013) and assessment for the area.

### Data collection

#### Study area

**Fig. 1:**
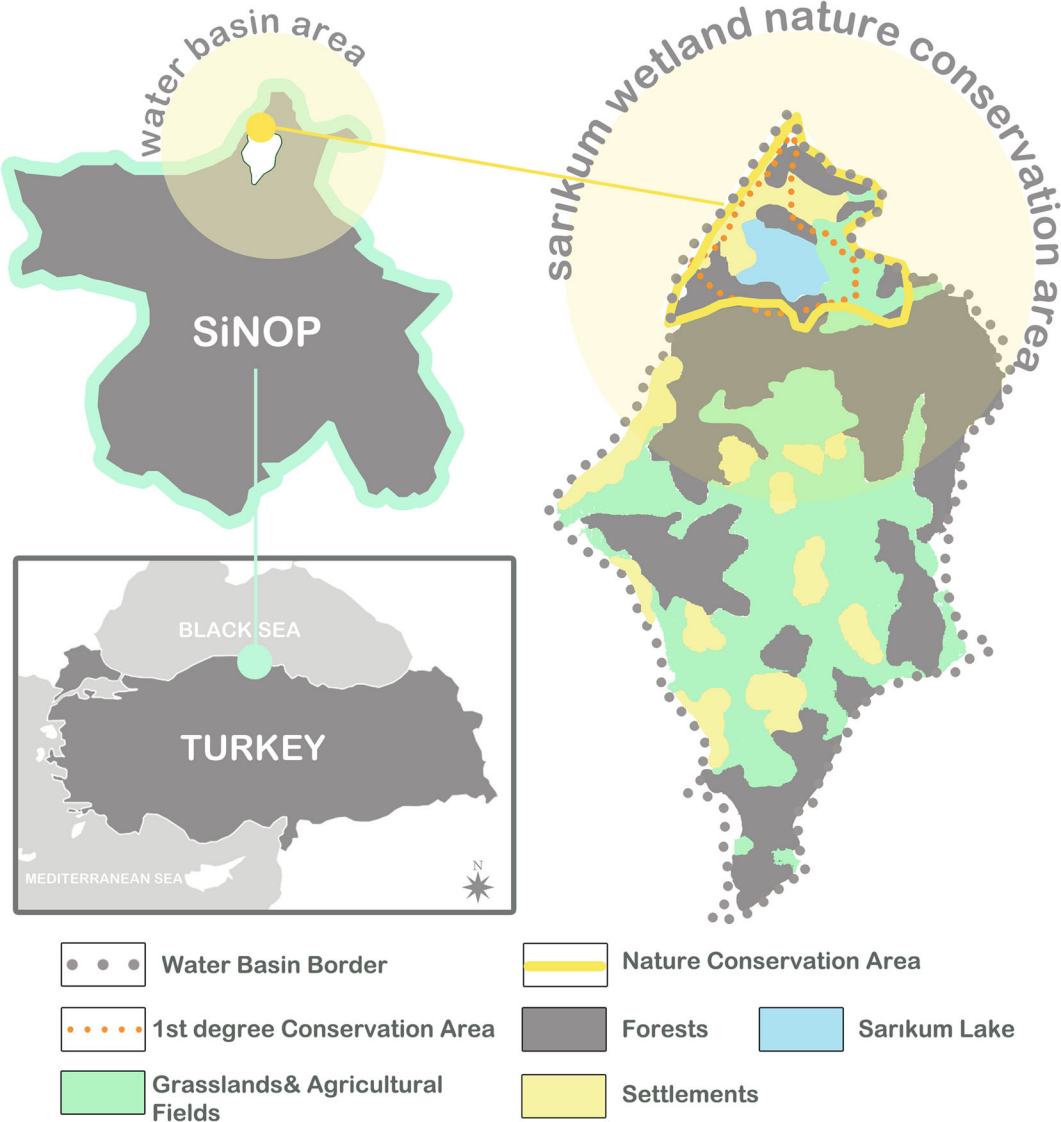
Position of research areas

Data was gathered regarding the SNPA which is located in the province of Sinop. Sarikum and its surrounding areas were declared as a I. Degree Natural Protected Area in 1981 and as a “Nature Protection Area” in 1987 (Sivaci et al. 2008; National Environmental Action Plan 1997). It is also a candidate wetland area for the Ramsar Agreement. It covers an area of 92.652 ha and includes a lagoon, a forest area approximately 500 ha, a I. Degree Natural Protected Area 53.170 ha, and a III. Degree Natural Protected Area 2.558 ha (National Environmental Action Plan 1997) (Fig. 1).

The area comprises wet and semi-wet areas, and has a warm winter and hot summer climate. It includes five main habitat types: sand dunes, inland surface waters, marshes, brushwood, and pastures. The climate and diversity of habitats have led to high biodiversity and species richness. The area and its surroundings are located on bird migration routes and are used by water birds, songbirds, and others for feeding and breeding (Yardim et al. 2008; Yilmaz 2005). Two hundred eighty-one bird species belonging to 58 families have been recorded in the area (Yardim et al. 2008). There are 9 herpetofauna species in the area and 33 mammal species belonging to 13 families. In addition, 12 species of zooplankton and 22 types of phytoplankton have been recorded (Sivaci et al. 2008). Regarding the plant flora of the basin in which the area is located, 522 species and sub-species belonging to 94 families have been recorded (Republic of Turkey Ministry of Forestry and Water Affairs 2012; Karaer 2003). There are 16 species endemic in this area. Some of these are the following: *Heracleum playtytaenium* Boiss., *Circium pseudopersonata* Boiss. & Bal. subsp. *Pseudopersonata*, *Taraxacum revertens* G. Hagl., *Danthus carmelitarum* Reut ex Boiss., *Verbascum bithynicum*, *Verbascum hereobarrbatu*, *Allium kastambulense* Kollman’dir (Sivaci et al. 2008).

Sarikum Village lies within the boundaries of the SNPA. It is part of the central county of Sinop and comprises 43 houses and a local population relying on agriculture and stockbreeding. Forestry, fishing, and tourism, though rare, also contribute to the local economy as alternative sources of income (Republic of Turkey Ministry of Forestry and Water Affairs 2012).

### R′WOT analysis

This study was based on information provided by relevant institutions, experts, NGOs, and the local population, and their inputs were prioritized in the strengths, weaknesses, opportunities, threats (SWOT) analyses. Our aim was to transform opportunities into advantages by strengthening weaknesses, while at the same time minimizing threats using strategies developed in the light of the data obtained and building on the potential of the area.

SWOT analysis is a mostly preferred technique while producing decisions about managements. This technique is used generally by outer and inner domains of associations, companies, or firms as a significant decision-making action (Kangas et al. 2003; Şahin and Yilmaz 2009; Nikolaou et al. 2011; Akbulak and Cengiz 2014). It is not only used in business sector but also is widely preferred in planning studies in order to reveal problems and potentials of an area and produce management strategies for that area. Nevertheless, by the fact that there is no question of setting up a relationship between this method’s criteria, evaluation and correlation of SWOT analysis results can be inefficient (Kurttila et al. 2000; Shinno et al. 2006). Thus, R′WOT analysis was performed using a combination of ranking analysis (RA) and linear combination analysis to measure and assess the SWOT factors identified for the SNPA area and to maximize the usefulness of the data collected (Yilmaz 2006; Öztürk and Tönük 2013). The R′WOT analysis comprised three stages. First, we gathered SWOT analyses. We asked the Sinop Provincial Directorate of Culture and Tourism, the Governor’s Office of Sinop, the Ministry of Forestry and Water Affairs, the 10th Regional Directorate, several nongovernmental organizations (NGOs), the Sarikum Village headman, and academicians at the Sinop University to each prepare their own SWOT analyses, which we assessed and analyzed.

In the second stage, 12 participants from four interest groups were interviewed and asked to make a comparison of the SWOT factors within each SWOT analysis and to say which issues were preferred (more important). The four groups were as follows: (1) the local government; (2) NGOs; (3) representative of the local people; and (4) expert groups. The 12 participants in the groups were as follows: (1) seven people from the local government (the Sinop Provincial Directorate of Culture and Tourism (one participant), the Governor’s Office of Sinop (one participant), the Sinop Provincial Special Administration (one participant), the Ministry of Forestry and Water Affairs, the 10th Regional Directorate, Nature Protection and Wetland Areas Branch Directorate (two participants), the Kastamonu Regional Directorate of Forest, the Sinop Directorate of Forest Management, the Sinop Provincial Directorate of Agriculture (two participants); (2) one from an NGO (the Sinop Society of Friends of Environment; (3) one representative of the local population (the headman of Sarikum Village); and (4) three sector experts from the Sinop University departments of biology and tourism. Nine ranks were included in the preference choices: 1 = weakly important, 3 = low importance, 5 = medium importance, 7 = very important, 9 = extremely important, with values of 2, 4, 6, and 8 being intermediate. The relative priority values of SWOT groups and of SWOT factors within the SWOT group were calculated based on the rank given to each SWOT group or SWOT factor. For example, assuming that a decision maker (*k*) gives a ranking to the SWOT factors in SWOT group (*j*) in the form of *r*_*jk1*_, *r*_*jk2*_,…, *r*_*jkm*_., the value of *X*_*ji*,_ the relative priority value of the SWOT factor (*i*) in SWOT group (*j*) may be calculated as follows, using RA:

$$ \begin{array}{ll}{x}_{ij}=\frac{{\displaystyle {\sum}_k{r}_{jki}}}{{\displaystyle {\sum}_k{\displaystyle {\sum}_k{r}_{jki}}}}\hfill & \left(i=1,2,\dots .\mathrm{m}\right)\hfill \end{array} $$

In the third stage, the relative priority value of each SWOT factor and the priority value of the SWOT group of this factor were multiplied in a linear combination analysis. This “linear combination” operation was performed mathematically, and the priority values of the SWOT factors were placed on the same scale and made comparable to one another (Yilmaz 2006). This operation was performed separately for each of the four SWOT groups. The linear equation used in this technique is as follows:

$$ {P}_{ji=}{W}_{ji}{X}_{ji} $$

Where *P*_*ji*_ is the final priority value of the SWOT factor (*i*) in the SWOT group (*j*), *W*_*ji*_ is the relative and final priority value of the SWOT group (*j*) in which the SWOT factor (*i*) is included, and *X*_*ji*_ is the relative priority value of the SWOT factor (*i*) in the SWOT group (*j*) (Yilmaz et al. 2009).

### Assessment

All of the data obtained in the last section of the study were assessed and strategies for were developed.

## Results

In this study, the 12 participants (the local administration (7), an NGO (1), a representative of the local people (1), and expert groups (3)) were consulted, and the views of the participants were given equal weight with respect to their judgment and their assessment of the relative priorities of the strengths and weaknesses identified in the SWOT analyses. The R′WOT values for the four SWOT groups are shown in Table 2. The priority values for the SWOT groups and the factors for each participant group were determined separately. According to Table 1, the greatest concern was afforded to the threat category of the SWOT groups (0.304). In addition, the threat categories were considered most pressing by the university expert groups and the NGO, the weaknesses were rated most highly by the central administration, and the opportunities were of most interest to the local population. The “area’s having rich biodiversity and endemic species existence” (0.054) were considered the area’s greatest strength. The NGOs (0.064) and the expert group (0.076) rated the view that “the area’s having different ecosystem existence” was the second most important strength. The data describing the biodiversity and habitat richness of the area supported this. The central administration rated “the area’s having uncorrupted, superior natural resource values” (0.051) most highly and the local population most favored the factor of “local people’s sympathy to ecotourism” (0.036). Recognition of the core values of the area by the central administration, which comprises the decision making groups is significant for the development of a balanced protection-usage approach. The fact that local people who are poor in economic terms consider ecotourism to be an alternative source of income is significant for implementing effective ecotourism strategies.

**Table 1:** R′WOT analyses for the Sarikum Nature Protection Area

SWOT groups		R′WOT factors	Participants priority
Strengths	0.213	Existence of different Ecosystems	0.050
		Rich biodiversity and existence of endemic species	**0.054**
		Untouched and superior natural resource values	0.042
		Sympathy of local people toward ecotourism	0.035
		Offering various opportunities for ecotourism	0.032
Weaknesses	0.243	Lack of legal regulation of ecotourism	**0.070**
		Shortage of infrastructure (transportation, sewerage system, waste management, agricultural infrastructure etc.)	**0.070**
		Insufficient Infrastructure for ecotourism including advertising, lodgings, guides	0.060
		Insufficient civil society enterprises	0.032
		Low economic level of nearby villagers	0.036
		Climatic characteristics	0.041
Opportunities	0.240	Popularity of ecotourism	0.045
		The fact that interest groups have begun to work cooperatively in the province	0.035
		Existence of other natural areas in the province of Sinop	**0.073**
		Road and air transport links	0.051
		The area’s having more than one protection status (a candidate for Ramsar)	0.035
Threats	**0.304**	Being close to an area where a nuclear power plant is planned	0.051
		Increasing aridity in flatplain forest.	0.023
		Possibility of corruption of the identity of local people as a result of tourism	0.041
		Insufficient audits (Poaching, uncontrolled tourism)	0.043
		Protection-usage imbalance due to lack of a management plan	**0.054**
		Pollution due to sea transport	0.040
		Existence of rural settlement within the SNPA,	0.029

Some entries inside the table are normally written but the numbers which are in the high priority factors are shown in bold

Generally speaking, among the weaknesses, factors such as the “lack of legal regulation of ecotourism” and the “shortage of infrastructure (transportation, sewerage system, waste management, agricultural infrastructure etc.)” (0.070) were given a high priority. Although the area has protected area status, it has no effective audit mechanism and it is important to introduce further legal protection. While those two factors were identified by the NGOs (0.061) as the most important problems, shortage of infrastructure (0.055) was considered to be the most important factor by the expert group. Problems associated with agricultural activities and waste management in Sarikum Village, which is located within the boundaries of the area, are significant issues for the natural habitats of the area.

In general, the most highly rated SWOT factor in the opportunities group is the “existence of other natural areas in the province of Sinop” (0.073). Sinop is a significant province in having a shore in the West Black Sea Region, a natural park, a natural monument, and acknowledged natural scenic beauty. NGOs (0.084) rated this factor the most highly. The local population rated the factor “popularity of ecotourism” (0.102) quite highly. This gives added support to the factor “sympathy of local people toward ecotourism,” one of the strength factors. Both factors were highly rated prioritized by local people. This suggests that local people will give support to projects and studies regarding ecotourism. The positive participation of local people in studies to develop ecotourism will clearly significantly benefit measures to protect the core values of the area (Agarwal 2001; Berkes 2007; Krause et al. 2013; Rai 2007; Ruiz-Mallén and Corbera 2013). Protection of the area with greater status and improved legal controls (0.035) was considered as the least important factor. We believe that this is because most interest groups believe that education to promote an understanding of the need for environmental protection can be effective, rather than relying on legal sanctions.

In general, when the threats are assessed, the factor “protection-usage imbalance arising from the lack of a management plan” (0.054) was identified as the most pressing factor. It was the most highly rated factor by the central management (0.064), expert (0.045), and NGO (0.061) groups. In order to provide an effective balance between protection and usage in the area, a management plan needs to be prepared effectively and a management organization needs to be established through participation with all the groups concerned. Lower level plans (e.g., for ecotourism strategy, habitat management, visitor management, etc.) can be prepared in more detail within the overall management plan to provide tools to achieve the sustainable management of natural resources. “Being close to the area determined for nuclear power plant construction” (0.054) is considered the most important threat by local people, who are clearly concerned about the planned construction of this plant.

The highest priority SWOT groups and factors of all participant groups are summarized in Table 2. This table shows that threats are more prior than the other SWOT group factors strengths, weakness, and opportunities.

**Table 2:** Highest priority SWOT factors of participant groups

Participant groups	Highest priority SWOT group	Highest priority factor/factors
Local Government	Weaknesses (0.282)	Insufficient Infrastructure for ecotourism including advertising, lodgings, guides (0.061)
Local People	Opportunities (03.75)	Popularity of ecotourism (0.102)
Expert Group	Threats (0.280)	Possibility of corruption of the identity of local people as a result of tourism (0.045)
		Protection-usage imbalance due to lack of a management plan (0.045)
		Existence of rural settlement within the SNPA (0.045)
NGO	Threats (0.375)	Being close to an area where a nuclear power plant is planned (0.061)
		Possibility of corruption of the identity of local people as a result of tourism (0.061)
		Insufficient audits (Poaching, uncontrolled tourism) (0.061)
		Protection-usage imbalance due to lack of a management plan (0.061)
		Pollution due to sea transport (0.061)
General	Threats (0.304)	Protection-usage imbalance due to lack of a management plan (0.040)

## Discussion

It is necessary to use existing resources more carefully owing to rapid population increase and the poor understating of ecology and conservation in Turkey (Öztürk and Ayan 2015). Wetland areas in particular have outstanding functions and values compared to other ecosystems. They play vital roles in regulating regional water regimes, softening the climate, and improving water quality by the retention of toxic substances, in addition to sustaining a rich wildlife, particularly of water birds. Furthermore, they contribute to the regional and national economy through the provision of fishing, hunting, rush processing, and tourism (Gündoğdu et al. 2005; Özcan et al. 2009).

All of the data we gathered indicate that the SNPA is rich in natural resources and a variety of different ecosystems, both aquatic (stagnant water and marshes) and terrestrial (forest, sand dune, ruderal, and segetal). In the tourism sector, where interest in natural areas increases gradually, it is important to establish a balance between protection and usage. Sinop is located in the west of the Black Sea Region and the occurrence of rainy months, and cold and snowy days in the province leads to peaks of tourism activity in certain months, particularly of activities based at sea. Nevertheless, the SNPA is a potentially important area for ecotourism, and could yield a significant source of alternative income for local people particularly if tourist activity can be developed over the full year and remain respectful of natural areas, and the identity and cultural values of the local population. Consequently, the area is ideal for many activities including ornithological and floristic observation, exploration walking, trekking, and photography. However, management plans that support species protection and conservation will be needed in specific areas where the ecosystems are sensitive to disturbance. More detailed plans, which are lower level parts of the greater management plan, such as visitor management plan, and habitat management plan were not prepared for the area. We imagine that this study will eventually guide the preparation of these lower level plans. The suggested strategies and applicability time in terms of short term, medium term, and long term of these strategies are shown in Table 3.

**Table 3:** Strategies and sub-strategies

Strategies and sub-strategies	Applicability time
Strategy 1: Provision of effective protection of natural resource values:	
- Create an integrated management plan with a participatory approach; instigate necessary training and education programs for all interest groups	***
-Integrate all the environmental information and core values using Geographical Information Systems (GIS) to create an information system and database to assist in establishing balances between the sensitive ecosystems	***
-Perform a detailed inventory of the flora of the area and develop protection programs for key species, focusing particularly on endemics included in the IUCN Red List categories and in the Bern Contract Appendix 1 (viz. *Crocus speciosus sp. Xantalamus* (sunflower) and *Isatis areneria* (sand dyer’s woad).	**
-Perform a detailed inventory of the fauna of the area, specifically of endangered birds and mammals (viz. *Lutra lutra* (sea otter), *Oxuyura loucocephala* (white headed duck) and zooplankton species including *Lecane punctata* and *Lecane hastata*); develop programs to protect those animals, creating observation areas for migrating birds, and identifying sites of special interest	**
-Provide effective audit mechanisms for conservation measures, particularly during the mating and reproductive periods of animal species	*
-Integrate all the environmental information and core values using Geographical Information Systems (GIS) to create an information system and database to assist in establishing balances between the sensitive ecosystems	*
-Create protected strips along the margins of water sources, especially those feeding the Longose forest areas, and make them off limits to visitors	*
Strategy 2: Development of infrastructure facilities, following the strategies listed below:	
-Create an ecotourism infrastructure, including picnic areas, overnight accommodation, museums, botanical gardens, zoos, visitor information centers, and sales outlets outside the protected area (i.e., around Sarikum Village),	***
-Organize courses for local people to learn to be nature guides and to provide visitor accommodation	**
-Produce promotional and information tools, such as internet sites and journals to foster national and international recognition of the area	*
-Strengthen transport networks and provide alternative means of transportation (e.g., by the seaway)	*
-Establish solid waste disposal facilities throughout the province of Sinop	***
-Prepare and complete projects for the urban waste water refinery system in the province	*
-Stabilize sand dunes and carry out the studies necessary to prevent them being a threat to village people	*
-Construct an agricultural infrastructure, drainage ducts, and disinfection pools in areas close to village settlements	**
-Establish an effective rainwater drainage system	*
-Make regular measurements of surface- and groundwater quality, and increase water monitoring and auditing studies	*
-Broadcast the results of studies into the short- and long-term effects of the planned nuclear power plant	*

*refers to short term; ** refers to medium term; *** refers to long term

## Conclusions

Maintaining the sustainability of all living things in areas with sensitive ecosystems requires a reasonable level of management action. Preparation of upper level management plans requires a well-disciplined study team. Consequently, this participatory study solicited SWOT analyses from all the interested parties and then performed a R′WOT analysis on them. This enabled the ranking of strategies concerned with ecological issues. Moreover, strategies created by combining the opinions of interest groups with the resource values of the area prioritize environmental protection and support economic development. Furthermore, strategies that target sustainable management of natural resources and socioeconomic development provide a road map for upper level resource management.

Once the data collected have been evaluated and a prioritized management plan have been developed, it is vital to review the country’s complex legal provisions for protection and to add and simplify laws as required.
